# Effective Melanin Depigmentation of Human and Murine Ocular Tissues: An Improved Method for Paraffin and Frozen Sections

**DOI:** 10.1371/journal.pone.0102512

**Published:** 2014-07-15

**Authors:** Caroline Manicam, Susanne Pitz, Christoph Brochhausen, Franz H. Grus, Norbert Pfeiffer, Adrian Gericke

**Affiliations:** 1 Department of Ophthalmology, University Medical Center, Johannes Gutenberg University Mainz, Germany; 2 Institute of Pathology, University Medical Center, Johannes Gutenberg University Mainz, Germany; The Chinese University of Hong Kong, Hong Kong

## Abstract

**Purpose:**

The removal of excessive melanin pigments that obscure ocular tissue morphology is important to address scientific questions and for differential diagnosis of ocular tumours based on histology. Thus, the goal of the present study was to establish an effective and fast melanin bleaching method for paraffin and frozen mouse and human ocular tissues.

**Methods:**

Paraffin-embedded and frozen ocular specimens from mice and human donors were subjected to bleaching employing two methods. The first employed potassium permanganate (KMnO_4_) with oxalic acid, and the second 10% hydrogen peroxide (H_2_O_2_). To determine optimal bleaching conditions, depigmentation was carried out at various incubation times. The effect of diluents used for 10% H_2_O_2_ was assessed using phosphate-buffered saline (PBS), and deionized water. Three different slide types and two fixatives, which were ice-cold acetone with 80% methanol, and 4% paraformaldehyde (PFA) were used to determine the optimal conditions for better tissue adherence during bleaching. All tissues were stained in hematoxylin and eosin for histological evaluation.

**Results:**

Optimal bleaching was achieved using warm 10% H_2_O_2_ diluted in PBS at 65°C for 120 minutes. Chromium-gelatin-coated slides prevented tissue detachment. Adherence of cryosections was also improved with post-fixation using 4% PFA and overnight air-drying at RT after cryosectioning. Tissue morphology was preserved under these conditions. Conversely, tissues bleached in KMnO_4_/oxalic acid demonstrated poor depigmentation with extensive tissue damage.

**Conclusions:**

Warm dilute H_2_O_2_ at 65°C for 120 minutes rapidly and effectively bleached both cryo- and paraffin sections of murine and human ocular tissues.

## Introduction

The eye is a specialized organ that comprises several layers of melanin-pigmented tissues with differing embryonic origins. Melanocytes in the ocular tissue are localized in the uvea, retinal pigment epithelium and surface epithelia, with heavy pigmentation concentrated in the uvea [1]. The melanin-forming cells in the uveal tract develop from the neural crest, which is also the origin of hair and skin melanocytes [2]. Melanin is a naturally occurring complex oligometric material that consists of various monomers, which are the oxidation and tautomerization products of DOPA (dihydroxyphenylalanine) derived from the amino acid tyrosine [3], [4]. This pigment is characteristically brown-black (eumelanin) and the difference in melanization found in various regions of the eye has been attributed to the specific functional relevance of melanin [5].

Bleaching techniques are important to remove excessive melanin granules that obscure the morphology of tissues subsequently interfering with differential diagnosis. This is especially important in the diagnosis and prognosis of melanomas, in which heavily pigmented tissues mask the interpretation of malignancy in histological sections [6]. Melanoma is the most common primary malignancy of the eye [7], and the use of melanin bleaching is indispensable in the interpretation of richly pigmented melanocytic lesions.

Melanin bleaching techniques have emerged as early as 1897 when Alfieri introduced the traditional oxidation method employing 0.1% potassium permanganate [8]. Only two methods are still widely used for histopathological studies. The classic bleaching technique employing dilute hydrogen peroxide (H_2_O_2_) and potassium permanganate (KMnO_4_) followed by oxalic acid treatment are highly preferred due to their effectiveness in removing the pigments and the easy incorporation into the conventional staining protocols [4], [9]. Both methods, which take advantage of the characteristic of melanin solubility in alkaline solutions have merits as well as drawbacks in certain circumstances [4]. Despite the emergence of many studies reporting on the efficacy of both methods, there are still limitations in terms of long bleaching time and effectiveness of the agents at various conditions. Moreover, most bleaching studies used either paraffin-embedded or frozen tissues. Therefore, the optimization of various factors involved in the depigmentation procedure is critical to determine the best outcome.

The purpose of the present study is to establish a rapid and effective melanin bleaching method in both cryo- and paraffin-embedded ocular tissues of human and murine origin using KMnO_4_/oxalic acid and dilute H_2_O_2._

## Materials and Methods

### Tissue samples

All animal studies conformed to the ARVO Statement for the Use of Animals in Ophthalmic and Vision Research, and were approved by the Animal Care Committee of Rhineland-Palatinate, Germany. Male C57BL/6J mice were sacrificed by carbondioxide inhalation and their eyes were rapidly enucleated and placed in OCT cryomedia (Tissue Tek, Sakura Finetek Europe, Netherlands) in a sagittal horizontal orientation and immediately frozen at –20°C in a freezer. Serial sections were cut at 8 µm thickness and adhered onto glass microscope slides. Some eyes from male mice of the C57BL/6J background were also subjected to fixation in freshly prepared 4% paraformaldehyde (PFA) in 0.1 M phosphate buffer saline (PBS, pH 7.4) for 24 hours, processed and embedded in paraffin wax. The paraffin sections were cut at 5 µm thickness and mounted onto glass slides.

Human iris and uveal melanoma samples were obtained from patients, who underwent enucleation at the Department of Ophthalmology, University Medical Center Mainz. Informed written consent was obtained from all patients prior to surgery. Approval from the ethics committee of Rhineland-Palatinate was obtained in advance. The acquisition and use of human tissues for experimental purposes conformed to the tenets of the Declaration of Helsinki. The tissues have been fixed in freshly prepared 4% paraformaldehyde in 0.1 M phosphate buffer (pH 7.4) for 24 hours prior to routine processing and embedded in paraffin. Sections were cut at 5 µm thickness and mounted onto glass microscope slides.

### Tissue adherence on microscopic slides

Tissue sections were adhered onto three types of microscopic slides, which were the normal uncoated (Diagonal GmbH & Co., Münster, Germany), Superfrost Plus (Thermo Scientific, Gerhard Menzel GmbH, Braunschweig, Germany), and subbed (chromium-gelatin coated) slides. The subbed slides were prepared according to a method described elsewhere [10] with modifications. Briefly, 0.5 g gelatin from bovine skin (type Bloom 225, Sigma-Aldrich, St. Louis, MO, USA) was dissolved in 100 ml of deionized water at 70°C while stirring. After the gelatin has dissolved and cooled to room temperature (RT), 0.05 g chromium (III) potassium sulphate dodecahydrate (VWR International, Leuven, Belgium) was added to the solution. This solution can be stored at 4°C up to several weeks and should be filtered immediately before use. Clean glass slides were dipped into this chromium-gelatin solution for a few seconds, excess blotted onto filter paper, and dried for 48 h at RT. Dried slides can be stored in dust-free, clean slide boxes at RT until further use.

**Figure 1:**
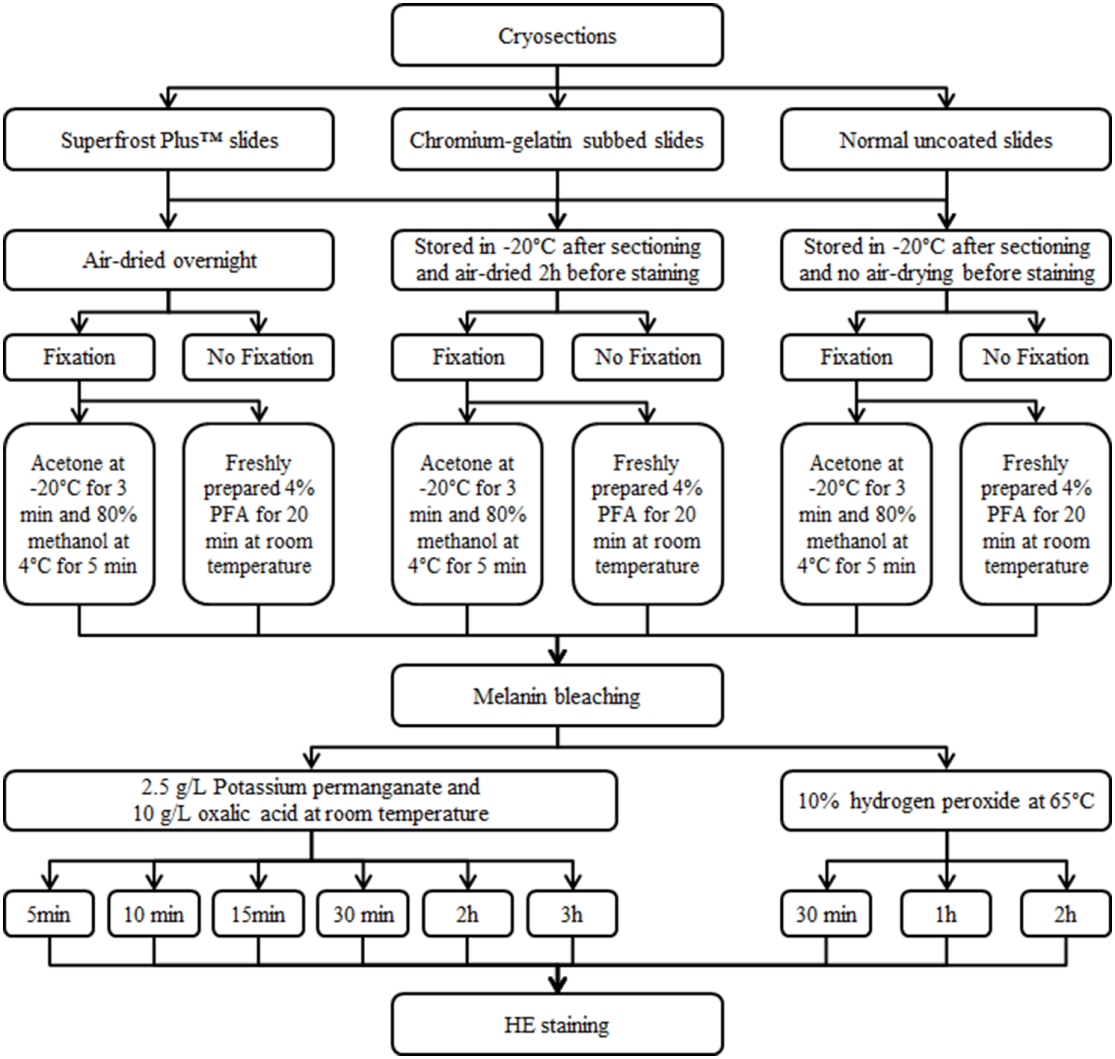
Flow-chart representing fixation and bleaching methods employed for frozen tissue samples.

Paraffin-embedded tissue sections were dried in a dry oven at 37°C overnight after sectioning and at 60°C for 30 minutes prior to bleaching. Frozen tissue sections were subjected to air-drying overnight after cryosectioning or stored in –20°C after sectioning and air-dried 2 h before bleaching. A control group that was not air-dried after sectioning and before bleaching was prepared for comparison of the effect of air-drying on tissue adherence. Figure 1 shows the flow-chart for the different parameters that were optimized for the frozen tissue specimens.

### Melanin bleaching

Melanin bleaching was carried out using two most widely employed methods, potassium permanganate and H_2_O_2._ Different parameters were evaluated to determine the optimum conditions for reproducible and efficient bleaching of ocular tissues.

### KMnO_4_/oxalic acid

The ocular tissue bleaching procedure employing potassium permanganate was carried out according to two studies described previously [11], [12]. Briefly, tissues sections were exposed to treatment with 2.5 g/L potassium permanganate for 5, 10, 15, 30, 120 and 180 min followed by 10 g/L oxalic acid for 5 min at RT.

### Dilute H_2_O_2_

A modified melanin bleaching technique using warm dilute H_2_O_2_ (10%) was employed according to studies described elsewhere [9], [13]. Various bleaching conditions for optimal depigmentation using H_2_O_2_ were assessed as follows.

### Assessment of optimal fixative

Two tissue fixation methods for post-fixation of cryosections were used and fixed tissues were compared to unfixed controls. The first batch of tissues were fixed in acetone at –20°C for 3 min and 80% methanol at 4°C for 5 min. The second group was fixed in freshly prepared 4% PFA for 20 min at room temperature (RT).

### Assessment of optimal diluents

The use of PBS (0.05 M, pH 7.4) and deionized water as diluents for H_2_O_2_ was evaluated to determine the optimal diluent for bleaching. In brief, 30% of H_2_O_2_ (Carl-Roth, Karlsruhe, Germany) was diluted with both diluents to a 10% solution. Paraffin-embedded tissue sections were dewaxed in three changes of xylene, rehydrated in graded alcohol and rinsed in deionized water for 2 minutes. Cryosections were washed in deionized water two times for 5 min each wash after fixation. Slides were then immersed in 10% H_2_O_2_ solution in a glass Coplin jar and placed in a pre-heated 65°C dry oven.

### Assessment of optimal temperature and incubation time

Previous studies demonstrated that elevated temperature provides a rapid bleaching effect employing dilute H_2_O_2_ [9], [13]_._ In view of this, both paraffin-embedded and frozen tissue sections were bleached in 10% H_2_O_2_ at 65°C in a dry oven for 30, 60, and 120 min.

### Hematoxylin and eosin staining after bleaching

Tissue sections were rinsed in deionized water for 10 min after the bleaching procedure and stained in Mayer’s hemalaun solution (AppliChem GmbH, Darmstadt, Germany) for 3 min, followed by a washing step under running tap water until excess color stopped bleeding. Slides were dipped in Scott’s bluing solution until the tissues started to turn blue, and rinsed in deionized water for 3 min. The use of 2% acid alcohol after hematoxylin was omitted in this method due to the progressive nature of the hematoxylin employed. If a regressive hematoxylin type, e.g., Harris’ hematoxylin is used, decolorization of excess stain is recommendable using acid alcohol. Prior to counterstaining with alcoholic Eosin Y (Sigma Aldrich, St. Louis, MO, USA) for 4 min, the tissue sections were placed in 95% ethanol for 1 min for differentiation and enhancement of the different hues of eosin. Subsequently, tissues were dehydrated in ascending concentrations of ethanol, cleared through three changes of xylene, mounted and cover-slipped. All specimens were stained in HE and bleached sections were compared to their unbleached counterparts.

## Results

### Melanin bleaching using potassium permanganate/oxalic acid

**Figure 2:**
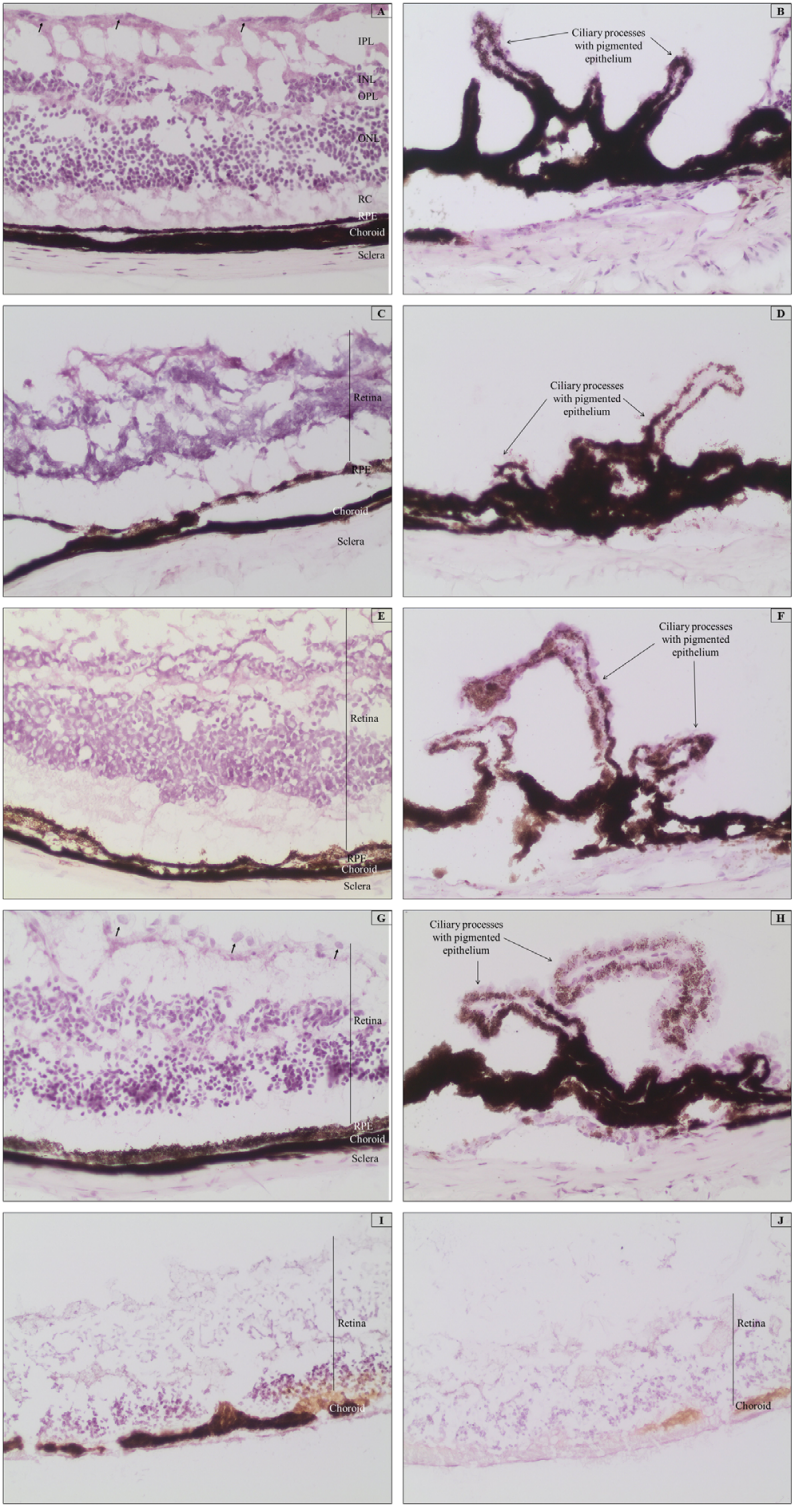
Representative photomicrographs of paraffin-embedded mouse ocular tissues treated with KMnO4 and oxalic acid. All tissues were routinely stained with HE. The degree of tissue disintegration increases with bleaching time and is especially evident at 120 and 180(A) Retinal tissue bleached for 5 min. (B) Ciliary processes bleached for 5 min. (C) Retinal tissue bleached for 10 min. (D) Ciliary processes bleached for 10 min. (E) Retinal tissue bleached for 15 min. (F) Ciliary processes bleached for 15 min. (G) Retinal tissue bleached for 30 min. (H) Ciliary processes bleached for 30 min. (I) Retinal tissue bleached for 120 min. (J) Retinal tissue bleached for 180 min. Magnification 200x. C: Choroid; RPE: Retinal pigmented epithelium; RC: Rods and cones; ONL: Outer nuclear layer; OPL: Outer plexiform layer; INL: Inner nuclear layer; IPL: Inner plexiform layer; Arrows indicate retinal ganglion cells.

Paraffin-embedded tissues subjected to melanin bleaching using 2.5 g/L potassium permanganate and 10 g/L oxalic acid for different times demonstrated no removal of the pigment and extensive tissue loss. Figure 2 show the photomicrographs of the retina and ciliary processes of mouse ocular tissues bleached with KMnO_4_/oxalic acid for 5, 10, 15, 30, 120, and 180 min at RT. Tissue deterioration was evident even after 5 minutes of bleaching (Figure 2A and B), and with prolonged bleaching time tissue morphology disintegrated. At 120 and 180 min of bleaching, the tissues were evidently damaged and only slight or no depigmentation was observed, as shown in Figure 2I and J. Similar results were observed in cryosections (data not shown).

### Melanin bleaching using dilute hydrogen peroxide

**Figure 3:**
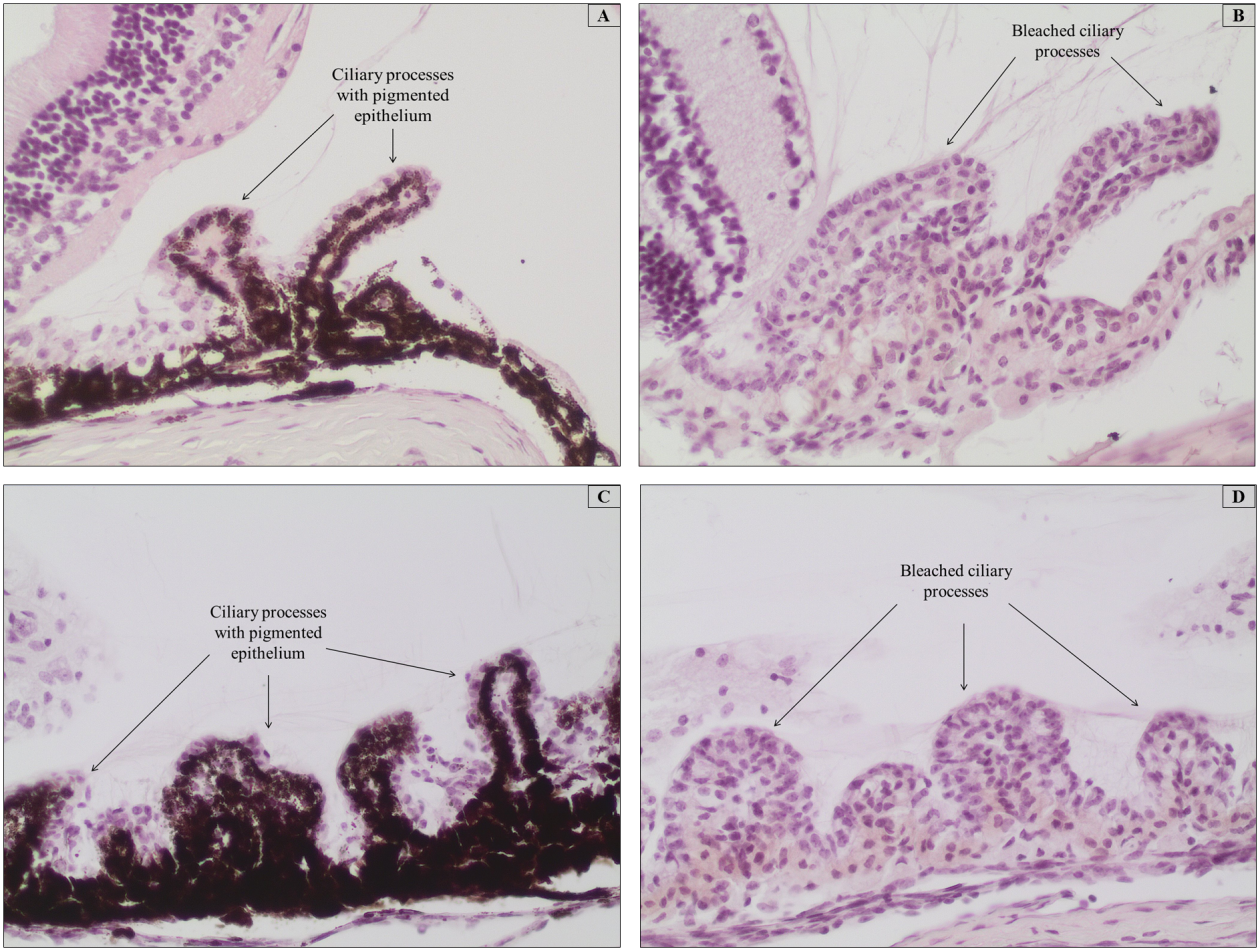
Representative photomicrographs of paraffin and frozen mouse ocular tissues treated with warm 10% H_2_O_2_ diluted in PBS at 65°C for 120 min compared with their respective controls. All tissues were routinely stained with HE. (A and C) Unbleached control tissues showing the heavily pigmented ciliary processes from the frozen and paraffin-embedded specimens, respectively. (B and D) Melanin-depigmented ocular tissues from the frozen and paraffin-embedded specimens, respectively. Bleaching was effective at these conditions and the tissue architecture is well-maintained after the treatment. Magnification 200x.

Frozen and paraffin-embedded ocular tissue samples of murine origin bleached with 10% H_2_O_2_ showed a complete bleaching effect in all tissues, such as in the ciliary body represented in Figure 3. The use of chromium-gelatin-coated slides ensured improved tissue adhesion to the slides, whereas Superfrost Plus and regular uncoated slides failed to prevent the tissue sections from falling off the slides. This was especially true for cryosections that were prone to detach from the slides during the vigorous bleaching treatment. Frozen tissue sections that were air-dried overnight had better adherence quality when compared to sections that were frozen at –20°C following sectioning and thawed for 2 hours at RT prior to bleaching and staining. Cryosections that were not air-dried after freezing at –20°C and immediately subjected to the depigmentation procedures had the poorest tissue quality in terms of tissue morphology preservation and adherence.

### Optimal fixation agent

**Figure 4:**
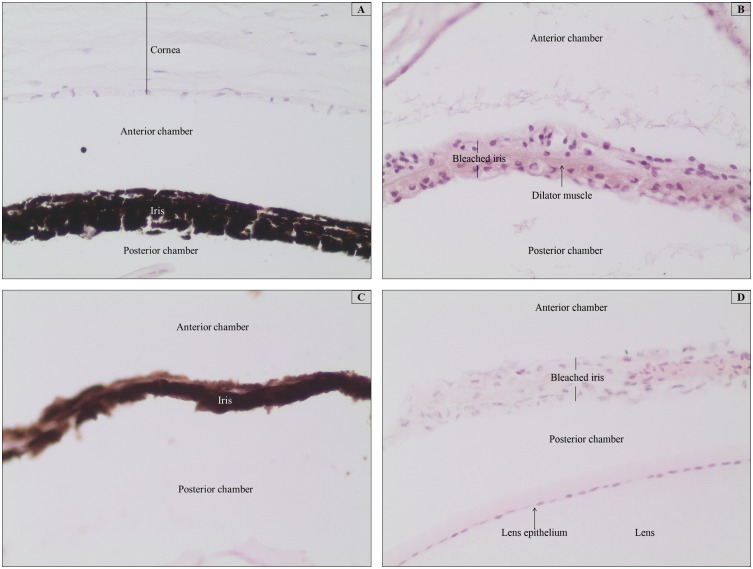
Photomicrographs of frozen and paraffin-embedded mouse ocular tissues that were fixed in freshly prepared 4% PFA prior to sectioning showing the iris dilator muscles. Bleaching was performed by treatment with warm 10% H_2_O_2_ diluted in PBS at 65°C for 120 min. All tissues were stained with HE. (A and C) Unbleached control tissues from the frozen and paraffin-embedded specimens, respectively. Dense pigmentation that obscures the visualization of the muscle is shown. (B and D) Bleached tissues from the frozen and paraffin-embedded specimens, respectively. Complete removal of the melanin pigmentation can be seen in these tissues, with preserved cytological details. Magnification 200x.

Cryosections that were post-fixed in acetone at –20°C for 3 min followed by 80% methanol at 4°C for 5 min before bleaching displayed disrupted cellular architecture and did not adhere well onto the slides, even onto subbed slides (results not shown). However, frozen tissues that were fixed in freshly prepared 4% PFA for 20 min at RT showed comparatively good results (Figure 4A and B) to paraffin tissues that were fixed in 4% PFA prior to routine processing and embedding in wax (Figure 4C and D). These photomicrographs show one of the most delicate tissue structures in the ocular sections, which is the dilator muscle of the iris. These microstructures are very sensitive to harsh treatments with highly alkaline solutions, such as the bleaching agents employed in this study, and are easily disintegrated if proper fixation and adhesion methods are not used (personal observation).

### Optimal diluents

Two different diluents, which were PBS (0.05 M, pH 7.4) and deionized water, were used to dilute H_2_O_2._ Our bleaching results show that the use of PBS as a diluent was superior to that of deionized water. Figure 3B and D show the final outcome of bleaching with 10% H_2_O_2_ that was diluted in PBS, for frozen and paraffin-embedded sections, respectively.

### Optimal incubation time

**Figure 5:**
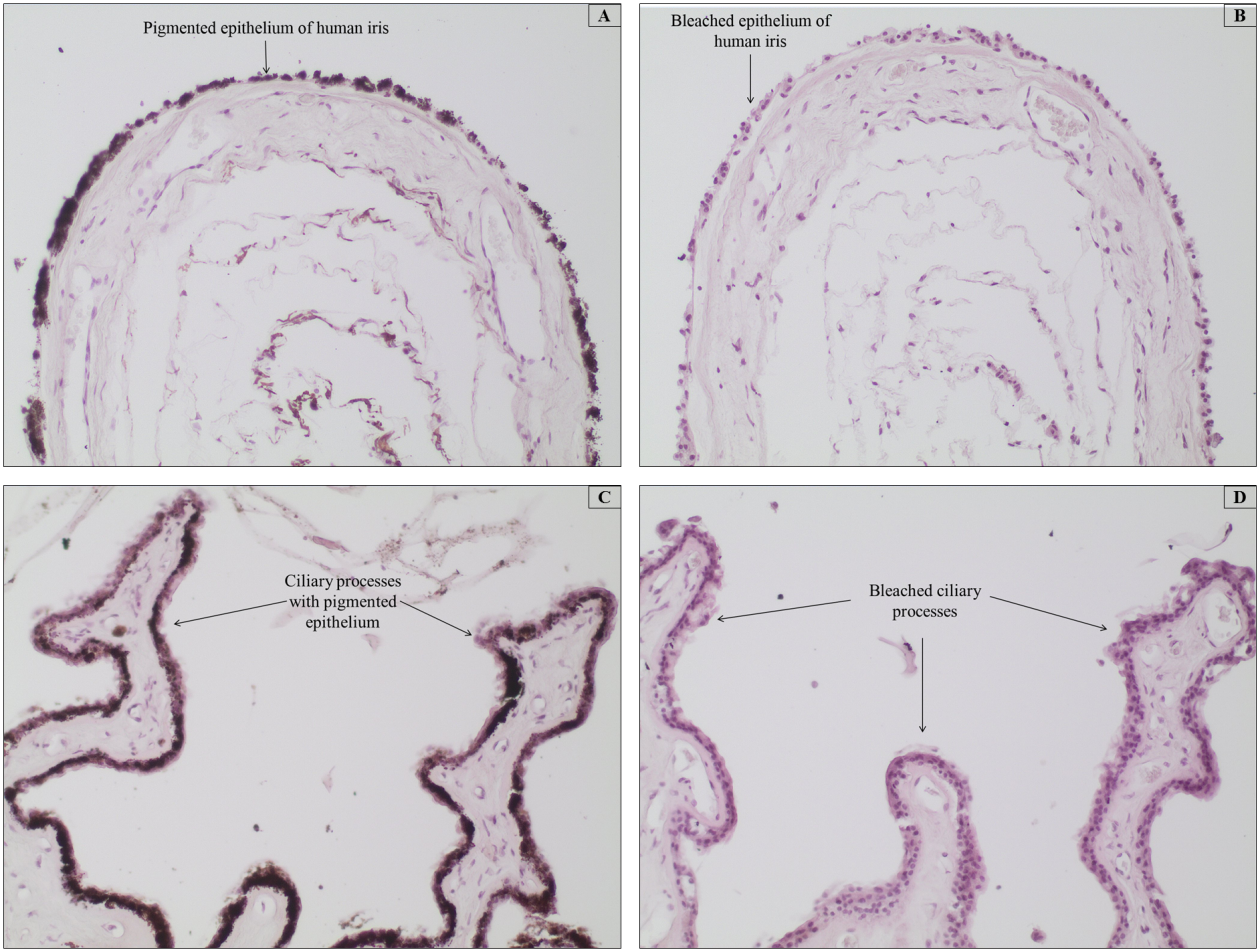
Paraffin-embedded human iris samples from healthy donors. Bleaching was performed by treatment with warm 10% H_2_O_2_ diluted in PBS at 65°C for 120 min. All tissues were stained with HE. (A and B) Photomicrographs show the unbleached and bleached pigment epithelium of the iris, respectively. (C and D) Photomicrographs of the ciliary processes are shown of both unbleached and bleached tissues, respectively. Magnification 200x.

**Figure 6:**
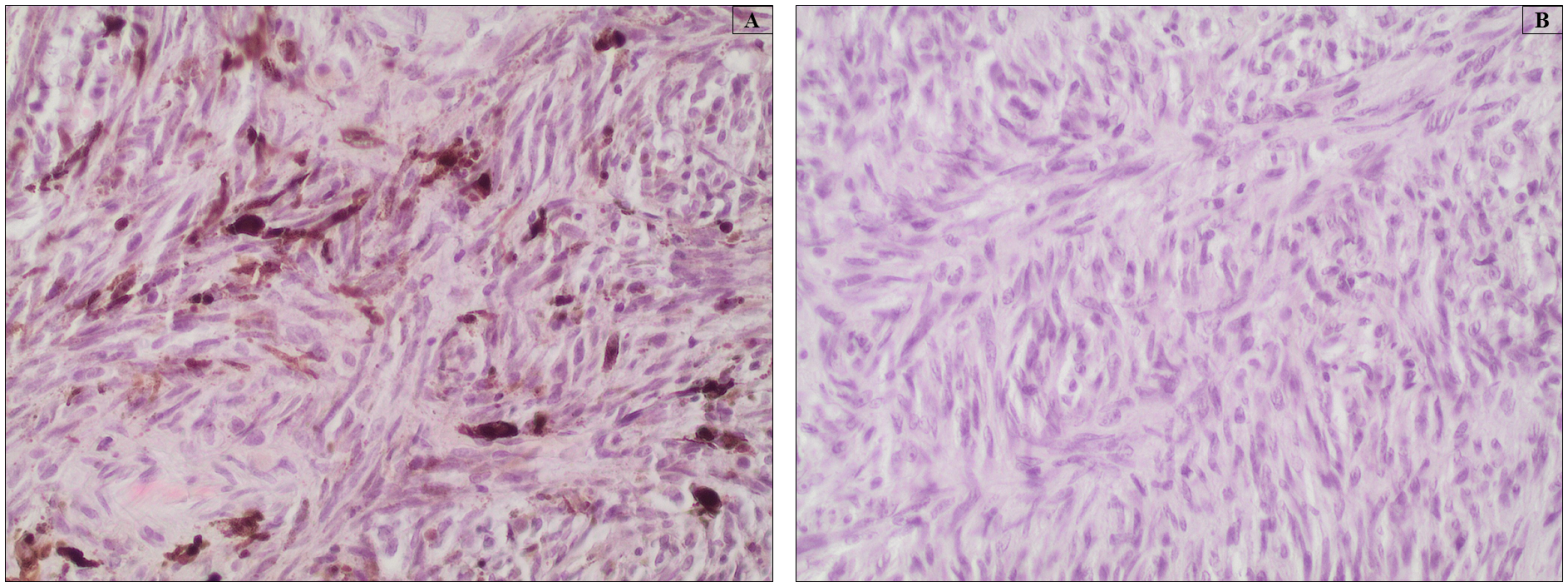
Paraffin-embedded tissue specimens representing human ocular melanoma. Bleaching was performed by treatment with warm 10% H_2_O_2_ diluted in PBS at 65°C for 120 min. All tissues were stained with HE. (A) Photomicrograph of unbleached tissue that shows the sporadic deposition of melanin pigments and typical spindle cellular morphology of melanoma characterized by elongated nuclei. (B) Photomicrograph of depigmented tissue shows complete removal of melanin from the pathologic tissue samples. Magnification 200x.

Optimum melanin depigmentation was achieved when tissue sections were treated with 10% H_2_O_2_ at 65°C for 120 minutes in a dry oven. The tissue morphology was well-preserved at these conditions for all tissue types, regardless of the degree of pigmentation. Bleaching was incomplete when tissues were incubated at the same temperature setting for 30 and 60 min (data not shown). This depigmentation temperature and time were also shown optimal for bleaching paraffin-embedded healthy and pathologic ocular tissues of human origin, as shown in Figure 5B and D, in comparison to their respective unbleached counterparts (Figure 5A and C). Pathologic samples that comprise of melanoma tissues with sporadic deposition of melanin were also completely bleached, while morphological details were well-preserved (Figure 6).

As with most protocols that involve multiple steps, there are a number of steps at which problems can occur that will compromise the desired end results. To identify the likely cause of problems, a brief troubleshooting guide is given in Table 1.

**Table 1:** Trouble-shooting guidelines for melanin bleaching using hydrogen peroxide (H_2_O_2_) and hematoxylin and eosin (HE) staining.

Problem areas	Trouble-shooting	Solutions
Tissues detaching from the slidesduring and after treatmentwith H_2_O_2_	a) H_2_O_2_ treatment is very harsh on tissues.Thus, slides need to be coated or subbed for better adherence.	a) Slide subbing with chromium gelatin.
	b) Under-fixation	b) Paraffin tissues should be fixed for an optimum period of time in 4% PFA prior to routine tissue processing. Cryosections should be post-fixed in 4% PFA before treatment with the bleaching agent.
Faint staining with eosin	a) Eosin is water-soluble. A washing stepwith water or diluted alcohol after eosin immersioncan remove the eosin from tissues.	a) Use anhydrous or 95% ethanol prior to counterstaining with eosin for enhancement and better differentiation of the stain.
	b) The pH of the eosin may be too alkalinei.e. above 5.0 due to a carryover ofthe bluing solution.	b) Wash the slides thoroughly after the bluing step (if one was used) to ensure there is no residual carryover.
Bleaching incomplete	Diluent for H_2_O_2_was deionized water.	Use PBS as diluents.
Over-staining with hematoxylin(the sections look too dark)	a) The tissue sections were stained toolong in regressive hematoxylin i.e. Harris’, Ehrlich’s,and Delafield’s.	a) Use an additional step of 2% acid alcohol to remove the excess stain after staining with hematoxylin.
	b) The differentiationstep was too short.	b) Adjust the differentiation time by dipping in acid alcohol until the slides start to turn pink.
Tissue sections are too pink	Pinkish color of the stained tissue sectionsis due to inadequate ‘bluing’.	Use a bluing agent e.g. tap water, Scott’s solution, ammonia solution in quick dipping motions of slides till they start to turn blue.

## Discussion

This is the first study presenting a rapid and effective melanin depigmentation method for both frozen and paraffin sections of ocular tissues. Dilute H_2_O_2_ proved superior to KMnO_4_/oxalic acid. Additionally, three important modifications enabled effective melanin depigmentation with superior tissue preservation. First, bleaching of the ocular tissues using 10% H_2_O_2_ at 65°C for 120 minutes was optimal for complete removal of the melanin pigments while preserving cytological details of both frozen and paraffin tissue specimens. It has been previously reported that successful melanin depigmentation methods using dilute H_2_O_2_, with particular interest on pathologic ocular tissues, usually take a minimum of 18 hours up to 2 or 3 weeks for thorough bleaching to occur at room temperature or at lower temperature i.e. 4°C [10], [14]–[17]. Nevertheless, longer bleaching time does not guarantee that all the pigment is completely removed from the tissues [18]. In the present study, we depigmented the tissues at 65°C instead of room or lower temperature, because a recent study by Liu et al [13] showed that elevating the temperature reduced the reaction time to 30 min in heavily pigmented paraffin-embedded non-ocular tissue samples. It is noteworthy that in our experiment though, 30 min was not sufficient for complete removal of the pigments. The differences in melanin composition between the non-ocular tissue samples used by Liu and the heavily pigmented ocular tissues used in the present study may explain these variations. Previously, Orchard et al [9] also demonstrated that 10% H_2_O_2_ bleaches best at 60°C for 150 minutes. This recent paradigm shift in increasing the temperature is due to efforts undertaken to address the long incubation time required for good bleaching without compromising the tissue integrity. On the other hand, higher temperatures exceeding 75°C may further shorten the bleaching time at the expense of tissue quality, which is not recommendable [13]. Effective bleaching was also achieved using 10% H_2_O_2_ that was diluted in PBS, compared to the use of deionized water as a diluent. Tissues that were bleached in water-diluted H_2_O_2_ showed the presence of residual melanin pigmentation and incomplete depigmentation patterns in all tissues, regardless of the tissue type. This finding is consistent with previous studies on non-ocular tissues that highlighted the use of a buffer as better diluent compared to distilled or deionized water [9], [13], [19].

Second, our investigation on the effect of fixative agents on tissue adherence as well as preservation of cyto-architecture showed that post-fixation of cryosections with 4% PFA gave the best outcome. This result is supported by the fact that formaldehyde, which is a cross-linking fixative, is characterized by its ability to form strong inter- and intramolecular covalent bonds between nitrogen molecules in gelatin used for subbing and tissue proteins, thus fixing the proteins *in situ* and preventing tissue detachment from slides [20], [21]. Conversely, fixation with ice-cold acetone and methanol did not improve tissue adherence, and the quality of sections was compromised. Although the exact mechanism of these coagulant fixatives is unknown, it has been proposed that they dehydrate the tissues, which ultimately results in disrupted protein conformations [21].

Our third finding demonstrated that the use of chromium gelatin-coated slides (subbed slides) significantly improved tissue adhesion to glass slides compared to commercially available, positively charged Superfrost Plus slides, which possess similar adhesion strength to uncoated slides, especially when undergoing bleaching. It is important to point out here that our results corroborate to similar problems encountered by other researchers utilizing the Superfrost Plus slides in the past [22]. It has also been recommended that subbed slides be utilized to ensure better tissue adhesion, particularly when bleaching ocular tissue using H_2_O_2_ [10]. Moreover, the use of chromium-gelatin as a coating medium has been claimed as the best compared to various other treatments [23]. On the other hand, air-drying the frozen tissue sections overnight at room temperature prevented tissue loss during the depigmentation procedure, compared to freezing and air-drying before treatment, and no air-drying. The tendency for tissues to detach from slides increases with shorter time of air-drying or absence of an air-drying step [22].

Our investigation was limited to conventional HE staining as the primary method of tissue examination after bleaching, and we did not embark on testing the effect of melanin bleaching on immunohistochemical staining protocols. However, numerous studies reported that bleaching with H_2_O_2_ did not interfere with the antigenicity of the tissues for a wide range of antibodies in both human and animal tissue samples [9], [24]–[27]. It is also of particular importance to indicate that all primary antibodies for various ophthalmopathologies have been stipulated to work well after H_2_O_2_ bleaching without prior testing in ocular tissues [10].

It is noteworthy, the outcome of bleaching using KMnO_4_/oxalic acid did not give promising results in our study, which is in agreement with the observations reported by Li et al [15]. The melanin granules were not thoroughly removed even after 120 to 180 min of bleaching, and tissue morphology preservation was compromised with prolonged bleaching time. Remarkably, a study reporting on melanin bleaching of the iris, choroid and ciliary body using 0.3% acidified potassium permanganate claimed that only 15 to 30 minutes is required for depigmentation to occur [12]. However, the degree of pigment removal and the integrity of the tissues following bleaching have not been clearly reported. Higher concentrations of KMnO_4_ were not employed in the present study considering previous reports demonstrating loss of tissue integrity and high susceptibility of section detachment from slides at higher KMnO_4_ concentrations [11], [28]. It was also documented that even at lower concentrations of KMnO_4_ (0.25% and 0.1%), tissue deterioration was evident [8]. On another note, there are also myriads of reports on the drawbacks of KMnO_4_ bleaching on immunostaining that hinder any further investigations [15], [29]. The latter bleaching agent has been shown to alter the specificity and sensitivity of antigenic epitopes of the tissues used for a variety of antibodies, apart from mediocre tissue conservation [30], [31]. Therefore, no further assessment was carried out employing this bleaching agent.

In conclusion, the findings of this study advocate the use of warm 10% H_2_O_2_ diluted in PBS at 65°C for 120 minutes for optimum, effective, and rapid removal of melanin pigments from both paraffin-embedded and frozen ocular tissue sections of murine and human origin. Judicious bleaching of ocular tissues for morphological assessment is pivotal in experimental procedures, for example, for accurate determination of uveal morphology. This method is also suitable for pathological tissues that require melanin depigmentation for morphological features that are often obscured, such as in the diagnosis of uveal melanoma. [6], [32] Cryosections are best air-dried overnight and post-fixed in 4% PFA prior to bleaching and staining in order to enhance tissue adherence and to preserve tissue architecture. The use of subbed slides, especially for cryosections, prevents the tissues from detaching from glass slides during vigorous treatment with highly alkaline solutions, such as H_2_O_2._ The techniques described in this article also provide sufficient details for reproducibility and therefore enhance the range of techniques available for investigations of ocular histology.
